# Myalgia and Hematuria in Association with Clonidine and Arginine Administration for Growth Hormone Stimulation Tests

**DOI:** 10.1155/2020/4827072

**Published:** 2020-05-26

**Authors:** Meghan Glibbery, Adam Fleming, Rahul Chanchlani, Olufemi Abiodun Ajani, Norma Marchetti, Alexa Marr, M. Constantine Samaan

**Affiliations:** ^1^Department of Pediatrics, McMaster University, Hamilton, Ontario, Canada; ^2^Division of Pediatric Endocrinology, McMaster Children's Hospital, Hamilton, Ontario, Canada; ^3^Michael G. De Groote School of Medicine, McMaster University, Hamilton, Ontario, Canada; ^4^Division of Pediatric Hematology/Oncology, McMaster Children's Hospital, Hamilton, Ontario, Canada; ^5^Division of Pediatric Nephrology, McMaster Children's Hospital, Hamilton, Ontario, Canada; ^6^Division of Neurosurgery, McMaster Children's Hospital, Hamilton, Ontario, Canada; ^7^Drug Information Centre, Hamilton Health Sciences, Hamilton, Ontario, Canada; ^8^Department of Pediatrics, University of Ottawa, Ottawa, Ontario, Canada; ^9^Department of Health Research Methods, Evidence and Impact, McMaster University, Hamilton, Ontario, Canada

## Abstract

Growth hormone deficiency (GHD) in children has significant impacts on growth and metabolism. Two-agent GH stimulation tests are commonly used to diagnose GHD, and these tests are generally considered safe. We report the case of a 5-year 5-month-old boy with a history of anaplastic ependymoma who underwent GH stimulation testing for growth deceleration using clonidine and arginine. He developed bilateral calf myalgia and gross hematuria within 24 hours of the tests. Myalgia and hematuria resolved spontaneously. Importantly, the literature review and database searches for hematuria identified 6 cases with clonidine and 20 cases with arginine. This case highlights an unusual combination of adverse reactions to clonidine and arginine in children undergoing GH stimulation testing to assess for GHD. Pediatric endocrinologists need to be aware of the potential for these side effects to allow appropriate management, and further studies are needed to clarify the mechanisms and frequency of these side effects. We recommend that patients and families need to be counselled about hematuria as an association of GH testing with these medications.

## 1. Introduction

Growth hormone (GH) testing is frequently used to assess children for growth hormone deficiency (GHD). One of the patient groups that are commonly tested includes survivors of childhood brain tumors. This is an emerging group of patients, thanks to advances in cancer care and treatments [1–3]. However, these children are at risk of GHD due to treatments that include surgery and cranial irradiation of >18 gray and with tumors located in the hypothalamic-pituitary region [4, 5].

Pediatric GHD can have significant impacts on growth and metabolic homeostasis. Diagnosing and treating GHD in children can prevent hypoglycemia in the early years of life and improve growth trajectories and body composition [6, 7]. Normally, GH secretion is pulsatile, and circulating levels are often low between pulses [8, 9]. Thus, provoked GH stimulation tests are a more reliable tool to assess GH secretory capacity than random testing. To enhance the accuracy of diagnosing GHD, it is accepted that responses to two GH stimulation tests are used to make the diagnosis [10, 11], and two of the commonly used agents in testing include clonidine and arginine [6]. These medications are generally accepted as being safe [12, 13].

We report an unusual combined side effect profile of myalgia and hematuria with clonidine and arginine stimulation testing in a pediatric brain tumor survivor.

## 2. Case Report

A 5-year 5-month-old male patient presented to the pediatric endocrinology clinic for assessment regarding growth deceleration. His history included a diagnosis of anaplastic ependymoma at one year of age, following a two-month history of vomiting, headaches, irritability, and abnormal gait. On magnetic resonance imaging, he had a large posterior fossa mass and underwent initial tumor debulking surgery with a near-total resection. The histopathology was consistent with a WHO Grade III anaplastic ependymoma.

The postoperative magnetic resonance imaging confirmed a small residual tumor in the floor of the fourth ventricle, and he was then treated as per the Children's Oncology Group (COG) study protocol ACNS 0831 [14, 15]. This protocol included two cycles of induction chemotherapy including vincristine, carboplatin, etoposide, and cyclophosphamide. Subsequent imaging suggested mild shrinkage of the tumor with some residual disease, and a second surgery was performed three months following the initial surgery that achieved gross total resection that was confirmed with postoperative magnetic resonance imaging.

Postoperatively, the patient developed left hemiparesis, facial palsy, and cranial nerve dysfunction that affected his swallowing. Following surgery, he received craniospiral irradiation at a dose of 54 gray given in 30 fractions over six weeks.

The patient was subsequently referred to the pediatric endocrinology clinic 30 months after completing treatment for concerns of short stature and reduced growth velocity. On evaluation, his height was 101 cm, which was below the 3^rd^ percentile for age and sex on the WHO growth chart, with a height *z*-score of −2.43. His growth velocity was 5 cm/year, which was between the 3^rd^–10^th^ percentiles on the growth velocity percentile chart [16]. His height dropped from between the 3^rd^–10^th^ percentile two years prior to presentation. This growth pattern was in the context of a midparental height of 178 cm, just above the 50^th^ percentile. His weight was 16.2 kg, which plotted on the 9^th^ percentile, and his body mass index z-score was 0.43. He was prepubertal and had left-sided hemiparesis. Based on his history of radiotherapy and growth deceleration, GHD was suspected, and he had GH testing.

The patient received the standard protocol for these tests at our institution, with clonidine administered at a dose of 100 *μ*g/m^2^ orally [17, 18] and L-arginine administered at a dose of 0.5 grams/kg in a 10% solution of normal saline infused intravenously over 30 minutes. Blood samples for growth hormone levels were collected at baseline, 30, 60, 90, and 120 minutes following the administration of these agents. GH peak response levels were normal and exceeded our institutional cutoffs of 8.0 *μ*g/L at 11.7 *μ*g/L and 20.8 *μ*g/L for clonidine and arginine, respectively. His blood pressure reading at baseline was 79/52 mmHg with a heart rate of 96 bpm. At 30, 60, and 90 minutes after clonidine administration, the blood pressure was 89/65, 74/43, and 83/48 mmHg with a heart rate of 114, 86, and 76 bpm, respectively. There were no measurements at 120 minutes. For arginine, the blood pressure at 30 minutes after the completion of the test was 73/40. He was given oral fluids, which he tolerated well and was discharged home.

He presented within 24 hours of completing these tests with significant bilateral calf muscle pain. The myalgia was most significant in the first 24 hours after GH test, preventing him from ambulating and weight bearing.

There were no rashes, redness, or local heat noted over the calves. A few hours after the onset of the myalgia, he developed gross painless hematuria. His urine was positive for +3 blood and +1 protein on point of care urinalysis, while urine microscopy reported gross blood in the sample with no red cell cast assessment. Urine nitrites were negative, while streptolysin O antibodies and renal ultrasound were normal. The creatinine was normal at 44 *μ*mol/L (normal 39–57) and in keeping with his pre-stimulation test creatinine level of 41 *μ*mol/L. His creatine kinase was 114 U/L (normal <200). The myalgia improved progressively the following day and fully resolved within one week, while the gross hematuria resolved by one week. Urinalysis was performed two weeks after GH testing to assess the resolution of hematuria, and at that point, the urine was negative for blood. During subsequent assessments in clinic over 11 months, he has had no further reported episodes of myalgia or hematuria.

## 3. Discussion

Growth hormone stimulation testing is the accepted standard of care for the evaluation of GHD in the pediatric population, and these tests are considered safe with appropriate monitoring [13]. In this report, we describe a rare combination of myalgia and gross painless hematuria that were related to the use of clonidine and arginine for GH stimulation testing.

The mechanisms through which GH stimulation agents act are diverse. While clonidine activates the *α*-adrenergic receptors to stimulate the secretion of GH-releasing hormone that will in turn stimulate GH secretion [19], arginine drives the suppression of somatostatin to allow the release of GH [20]. The potential side effects of clonidine include hypotension and dizziness, and young children may experience drowsiness, somnolence, and hypoglycemia [21]. The potential side effects of arginine include nausea, vomiting, numbness, headache, and venous irritation with rapid administration [22].

The bilateral calf myalgia in our patient is likely related to clonidine administration. However, while there are reports of myalgia with long-term clonidine use, there are no reports of bilateral calf pain or general myalgia in children following GH stimulation testing. The product monograph suggests that leg or muscle cramps may occur in 0.01–0.1% of cases following clonidine use. However, the mechanisms by which clonidine causes myalgia remain undetermined, and the monograph does not report if this side effect is encountered in pediatric patients [21]. An 8-week, randomized, double-blind, placebo-controlled trial in children and adolescents 6–17 years of age with ADHD reported the incidence of ‘pain in limb' following oral clonidine hydrochloride administration to be 2%. These patients received an extended-release clonidine hydrochloride preparation at a dose of 0.4 mg/day plus stimulants (methylphenidate or amphetamine) (*n* = 102) and were compared with patients who received only stimulants (*n* = 96) [23]. There are also published cases of lower limb myalgia following clonidine administration in the context of opioid withdrawal management in adults [24].

One possible mechanism by which clonidine may cause myalgia may be related to the acute onset of hypotension and bradycardia from its alpha-agonistic effects [13, 21], with reduced perfusion and transient ischemia of the calf muscles. It has been suggested that the use of the oral hydration therapy is one potential intervention to prevent hypotension with the test [25]. Our patient's blood pressure did drop by the end of the test, which may explain this clinical presentation. Alternatively, some drugs are known to induce a benign acute myositis, which produces a similar presentation of myalgia. However, neither clonidine nor arginine have previously been reported to cause acute myositis in the pediatric population [26], and none of the chemotherapeutic agents this patient received are suggested to cause long-term myalgia, especially that he was asymptomatic before the test and had a normal CK level after the test.

Hematuria has been reported as a rare side effect following arginine administration in the pediatric population [22]. A previous pediatric case series described three boys aged 6–11 years who experienced a benign, macroscopic hematuria following GH stimulation testing with clonidine and arginine hydrochloride that resolved within 3–4 days [27]. We further searched the FDA Adverse Events Reporting System (FAERS), a database that includes reports from healthcare professionals, consumers, and drug manufacturers. The FAERS database included 13 cases of arginine-linked hematuria. Eight of these cases were labelled as children, and five were of unknown age [28]. Only one female was reported in the latter group, while all other cases occurred in males. Importantly, the last reported case was from 2014; while some of the cases in the database may be related to the case reports noted in the literature, it is certain that some of the case reports are independent of the FAERS reports. Interestingly, the FAERS also reported 6 cases of hematuria with clonidine in pediatric male patients.

On reviewing the Canada Vigilance Adverse Reaction Online Database, four boys, two of whom were 12 years of age, while one was 11 years old and one with unspecified age, developed gross hematuria while undergoing GH testing with clonidine and arginine [29]. One additional case was reported of a 12-year-old boy on clonidine alone. The European Adverse Drug Formulary search did not yield any reports of arginine-related hematuria. The prevalence of micro- and macroscopic hematuria in children undergoing GH stimulation testing was measured in a study of 34 subjects undergoing GH stimulation testing using mainly clonidine and arginine [30]. Three subjects (all male; aged 9–12 years) developed hematuria that resolved spontaneously within 7 days of testing, which is congruent with our patient's history. These data provided an estimate of the prevalence of hematuria of 8.8%. Although the limited number of reports, small sample size, and the search of select databases are limitations, the data suggest that hematuria may be an underrecognized side effect of GH testing, and seem more common with arginine. Further studies are needed to clarify how common this association is [30].

The potential pathophysiology of arginine-related hematuria is poorly understood. It has been proposed that transient hypotension experienced by patients who received GH stimulation testing with both clonidine and arginine may cause nephritic changes and subsequent hematuria [30]. Although hematuria can be a marker of glomerular barrier damage, none of the patients in the aforementioned study or our patient had evidence of persistent renal disease. Future studies are needed to measure proximal tubular function to determine whether renal damage is actually occurring as a result of the arginine infusion [31, 32]. Of note, all cases of reported hematuria following GH stimulation testing thus far have been in male patients, which may potentially indicate a sex-specific effect of these drugs on the development of hematuria.

A unique component of our patient's medical history includes the anaplastic ependymoma with subsequent therapies including surgery and radiochemotherapy. Whether any effect of this therapy protocol could have contributed to the adverse reaction to the GH stimulation test is unknown. In particular, certain chemotherapeutic agents have been linked to long-term renal side-effects. Carboplatin, one of the chemotherapeutic drugs administered as part of our patient's treatment protocol, has demonstrated clinically important reductions in glomerular filtration rate in the adult population [33]. However, our patient exhibited no signs or abnormal investigations at the time of their treatment and afterwards that would indicate brain tumor therapy-driven renal injury.

## 4. Conclusion

This report highlights a rare combination of myalgia and hematuria associated with GH testing. While self-limiting and poorly understood from a mechanistic viewpoint, hematuria has been reported in at least 20 cases in the literature and databases and appears to be an underrecognized side effect of arginine. There are fewer cases of hematuria associated with clonidine.

This case emphasizes the need for pediatric endocrinologists performing GH testing with arginine and clonidine to be aware of these potential side effects, so that appropriate follow-up and management can be provided. Further studies are needed to determine the frequency of myalgia and hematuria in children undergoing GH testing and the exact mechanisms driving these adverse reactions. At this point, we recommend that patients and families need to be counselled about hematuria as an association of GH testing with these agents, and that hematuria is mainly related to arginine use.
